# Changes in microbial composition and interaction patterns of female urogenital tract and rectum in response to HPV infection

**DOI:** 10.1186/s12967-024-04916-2

**Published:** 2024-02-01

**Authors:** Yong-Hong Dong, Yu-Hua Luo, Chen-Jian Liu, Wen-Yu Huang, Lin Feng, Xing-Yuan Zou, Jin-Yan Zhou, Xiao-Ran Li

**Affiliations:** 1 Faculty of Life Science and Technology, Kunming University of Science and Technology, Kunming, 650500 Yunnan China; 2 Pediatrics Department, Yan’an Affiliated Hospital of Kunming Medical University, Kunming, 650051 Yunnan China; 3 Changchun Institute of Biological Products Co., Ltd., Changchun, 130012 Jilin China; 4 Guangdong Hybribio Biotech Co., Ltd., Chaozhou, 521000 Guangdong China

**Keywords:** HPV, Microbiota, Urogenital tract, Rectum, Cytokine

## Abstract

**Background:**

Previous studies have shown that changes in the microbial community of the female urogenital tract are associated with Human papillomavirus (HPV) infection. However, research on this association was mostly focused on a single site, and there are currently few joint studies on HPV infection and multiple sites in the female urogenital tract.

**Methods:**

We selected 102 healthy women from Yunnan Province as the research object, collected cervical exfoliation fluid, vaginal, urethral, and rectal swabs for microbial community analysis, and measured bacterial load, and related cytokine content. The link between HPV, microbiota, and inflammation was comprehensively evaluated using bioinformatics methods.

**Findings:**

The impact of HPV infection on the microbial composition of different parts varies. We have identified several signature bacterial genera that respond to HPV infection in several detection sites, such as *Corynebacterium*, *Lactobacillus*, *Campylobacter*, and *Cutibacterium* have been detected in multiple sites, reflecting their potential significance in cross body sites HPV infection responses. There was a solid microbial interaction network between the cervix, vagina, and urethra. The interrelationships between inflammatory factors and different bacterial genera might also affect the immune system's response to HPV infection.

**Interpretation:**

It might be an effective strategy to prevent and treat HPV infection by simultaneously understanding the correlation between the microbial changes in multiple parts of the female urogenital tract and rectum and HPV infection, and controlling the microbial network related to HPV infection in different parts.

**Supplementary Information:**

The online version contains supplementary material available at 10.1186/s12967-024-04916-2.

## Introduction

Human papillomavirus (HPV) is the most common sexually transmitted infection and a leading cause of genital warts and cervical cancer [1]. After the virus enters the human body, it integrates into the infected cells and initiate the transcriptional expression of oncoprotein (E6,E7), thus promoting the occurrence of cancer [2, 3]. Women's lifetime risk of HPV infection is about 80%, while the risk of eventually developing cervical cancer is 0.6% [4]. Most HPV will not cause disease on the human body after infection, though, most women will clear HPV within 12–24 months through the autoimmune mechanism, only a small part of these infections persist and may lead to precancerous lesions or cancer [5, 6]. HPV infection in women is caused by a number of factors, including sexual behavior, age of first sexual intercourse, number of sexual partners, condom use, smoking, etc. [7–9]. The female genital tract is protected against infections by a complex system composed of the mucosal epithelial barrier, the immune system, and a healthy microorganism producing lactic acid, hydrogen peroxide, halides, and antimicrobial peptides [10].

Studies have shown that cervicovaginal microbes may be involved in human immune response to HPV infection [11]. The imbalance of cervicovaginal microorganisms may promote the production of inflammatory factors (IL6, IL8, IL17), recruit immune cells such as antigen-presenting cells, change the cervicovaginal immune microenvironment, and establish the mechanism of viral infection. [12]. Compared to the gut microbiome, the female reproductive tract microbiome is relatively homogeneous [13, 14]. When vaginal *Lactobacillus* is absent and *G. vaginalis* and *Mobiluncus* spp. dominate, this is often accompanied by BV. [15]. HPV prevalence is influenced many factors, such as the composition and structure of vaginal microbiome and cytokines [16]. For example, *Lactobacillus* in cervicovaginal microbes have been reported to be associated with the clearance of high-risk HPV, *Gardnerella* was the dominant biomarker for HR-HPV progression [17]. Vaginal microbiota dominated by non-*Lactobacillus* species or *Lactobacillus iners* were associated with three to five times higher odds of any prevalent HPV *Lactobacillus crispatus* [18]. It is plausible that *Lactobacillus* disturb microbiome composition may lead to a pro-inflammatory environment increase malignant cell proliferation and HPV E6 and E7 oncogene expression [19]. Gut has a huge microbial system, which can interact with other organs, which is also one of the important factors affecting health outside the gut [20]. The anatomical proximity of the intestine and vagina allows for potential interaction between them [21]. Previous studies have found that specific patterns of intestinal bacteria are associated with reproductive tract lesions such as endometriosis and polycystic ovary syndrome [22, 23]. Therefore, changes in gut microbes may also be an important factor for affecting female reproductive tract health. The microbiome in the urinary tract is associated with the maintenance of health, and an unstable microbiome is associated with the development of urinary tract diseases, such as urinary tract infections (UTI) [24]. Studies have found common vaginal bacteria such as *Lactobacillus*, *Sneathia*, *Prevotella*, *Gardnerella*, *Atopobium*, *Peptoniphilus*, and *Finegoldia* are components of female urinary tract microbiota, and patients with urinary tract infection have higher *Gardnerella* and lower *Lactobacillus* load compared to non-urinary tract infected [25, 26]. At the same time, the female urethra is also the latent site of HPV [27]. Therefore, changes in the microenvironment of female urethra might also have effect on the reproductive tract, however at present, few studies have associated the female urethra microbiome with reproductive tract diseases [28, 29].

There is a microbiota continuum along the female reproductive tract, and the microorganisms in different parts may affect each other and even interfere with the occurrence and development of diseases [30]. In our previous study, common vaginal *Lactobacillus* and *Gardnerella* were detected in follicular fluid [31], and half of the placental microbial sequences in the environment where the fetus was conceived were detected in the mother's vaginal and rectal samples [32]. Within individuals, the genital microbiota can interact with other body sites, both proximal (such as the urinary tract) and distal (such as the rectum or oral cavity) [33]. Study has found that bacteria in the vagina can rise to colonize the uterus, grow and cause inflammation [34]. Common vaginal bacteria (such as *Lactobacillus*, *Sneathia*, *Prevotella*, *Gardnerella*) are components of the urinary tract microbiota in women [35–37]. The composition of vaginal microbes may be influenced by the entire urogenital tract. Our previous studies detected different proportions and types of HPV infection in cervical, vaginal, and rectal swabs, with different sites exhibiting different microbial changes under HPV infection [38]. The microbial communication patterns between the urogenital tract and other sites and the microbial networks they form may also affect HPV infection.

In this study, we hypothesized that microbial interactions between different urogenital tract sites in women may influence the colonization of HPV infection in the female body. At present, most studies only focused on the microenvironment changes of a single part of the female reproductive tract after HPV infection, while there was less research on the effect of solid microbial communication patterns between multiple parts of the reproductive tract on the clearance or persistence of HPV on the asymptomatic female. Therefore, it might be more meaningful to explore the influence of the microbial network between female urogenital tract and rectum on HPV infection. We analyzed and compared the composition and differences of urogenital and rectal microbes in asymptomatic female without HPV infection, as well as the microbiota of potential communication between different sites, and identified the microbiota associated with HPV infection. We provided novel insights into the mechanisms of microbial changes in the urogenital tract and rectum during HPV infection in female by building the connection with inflammatory factors.

## Materials and methods

### Sample collection and study design

We collected all samples, which sourced from 102 asymptomatic healthy women. This project was approved by the Ethics Committee of YAN’AN HOSPITAL of KUNMING CITY (2019-077-01). All subjects provided written informed consent without financial compensation. All samples were collected at the Obstetrics Department of YAN’AN HOSPITAL of KUNMING CITY in Yunnan Province, China. Women who met any of the following criteria were excluded: (1) They had used antibiotics or vaginal drugs in the past month; (2) Obvious cervical and vaginal symptoms; (3) Have a history of diabetes, autoimmune diseases, malignant tumors and other systemic diseases. Fourteen samples were collected per subject (3 repeated vaginal, urethral, and rectal swabs, 3 repeated cervical cell shedding fluid, and repeated blood samples). The survey included age, education, occupation, economic status, health habits, sexual activity, gynecological history, and knowledge of HPV. We collected cervical cell shedding fluid., which sourced from the subject's cervix and swabs from the vagina, urethra and rectum of female by medical professionals in the female examination room, and ensured that Cytobrush does not touch the participant's vaginal wall during the collection process for minimizing vaginal contamination. Intravenous blood collections followed. Specifically, swabs coated with sterile saline were placed into the posterior fornix of the vagina and 2–3 cm inside the rectum and urethra, and gently rotated for about 10 s. Cell samples and secretions from the inner surface of the uterine neck were gently scraped with a medical brush, and samples from each part were stored in a sterile compartment tube. The blood samples were centrifuged at 8,000 rpm for 10 min in a 4 ℃ cryogenic centrifuge for separating the serum. All samples were stored in a −80 ℃ refrigerator and transported back to the laboratory with refrigerant for further processing.

### Whole-genomic DNA extraction from swabs and PCR amplification and sequencing

DNA extraction was carried out in a strictly controlled sterile environment. Due to the low micro-biomass in the urogenital tract, we set up three repeated negative controls in each batch treatment to avoid incorporating false positive results into subsequent experiments. Four ml of cervical cell shedding fluid was taken and centrifuged for 10 min at a rate of 12,000 r/min. The centrifuged particles were used to DNA extraction. The microbial DNA of all samples was extracted using the QIAGEN Pro Prower fecal DNA Kit (Hilden, Germany) according to the kit instructions. DNA yield was assessed using Nanodrop 2000 (Thermo Fisher, USA). Primers for PCR amplification were 515F [39] and 909R [40]. To differentiate the samples after sequencing, all primers were used to add Illumina adapter sequences and double-indexed barcodes. Fifteen nanograms (ng) of DNA was used as the template in each PCR reaction, and the reaction conditions were as follows: pre-denaturation at 95 ℃ for 15 s, followed by cycling at 95 ℃ for 3 min for 30 cycles, annealing at 51 ℃ for 30 s, extension at 72 ℃ for 30 s, and a final extension step at 72 ℃ for 5 min. The same reagents and consumables were used during DNA extraction and PCR for each sample and the PCR amplification procedure was standardized for all DNA samples in the sequencing experiment, including the negative controls. No PCR bands were observed in the negative control samples. The PCR products were purified using the UltraClean PCR Cleaning up Kit (MOBIO, USA), and the equivalent PCR products were sequenced using the Illumina Miseq™ system (Illumina, USA).

### Bioinformatic analysis of 16S rRNA gene sequences

All sequences were processed using Mothur v.1.48.0 [41] according to the standard operating procedure described earlier. High quality sequences were obtained by removing sequences with ambiguous bases, with a low-quality read length, and chimeras identified using uchime [42]. Furthermore, we filtered mitochondria and chloroplasts. The obtained high-quality sequences were compared against the SILVA database (v138) [43] and the OTU was generated by using the cutoff value 0.03. We classified the sequence using the classify seqs command. In order to solve the problem of unequal sequencing depth, we have normalized the data for sampling depth of 10,000 sequences per sample for subsequent analysis. Alpha diversity (i.e., ACE, Chao1, Shannon, Invsimpson diversity) was calculated using the Mothur software. The between-samples beta difference was evaluated with the principal coordinates analysis (PCoA) using Mothur, enabling the projection of each sample and the variable loadings of OTU onto individual principal components (PCs). Permutational multivariate analysis of variance (PERMANOVA) results were shown variation in the composition of microbial communities in different groups. Projection to latent structure discriminant analysis (PLS-DA) was used to differentiate the HPV positive (HPV-P) and HPV negative (HPV-N) [44]. The metabolites were screened by the variable importance in the project (VIP) > 1 in the PLS-DA model. The Spearman was performed to identify the correlation (correlation p < 0.05 and |rho|> 0.6). The generated co-occurrence network was visualized in Cytoscape (v3.9.1) and subnetworks were extracted using the MCODE plug-in. The potential sources of vaginal microbiota was predicted by SourceTracker (version0.9.5) [45]. The metagenomes predicted by PICRUSt2 and MetaCyc Metabolic Pathway Database (http://metacyc.org/) revealed the microbial contribution to metabolites.

### Statistical analysis of demographic data

Data analysis was performed using R software (version 4.1.1). The chi-square test or Fisher's exact test was utilized to compare categorical variables, and the results were presented as frequency and percentage. Continuous variables were expressed as mean ± standard error of mean. To assess significant differences in the α-diversity index within each group, the Wilcoxon rank sum test was employed to calculate diversity differences, followed by Dunn's multiple comparison test. Functional pathways with significant differences were identified using the linear discriminant analysis effect size (LDA score > 2) algorithm [46]. The random forest model was obtained using an R package, and the ROC curve was plotted using the pROC package. Internal validation was conducted through tenfold cross-validation. Mantel correlations between microbial compositions and inflammatory factor data were calculated based on UniFrac distance using the R software package (9,999 permutations).

### Quantification of bacteria and *Lactobacillus*

The copy numbers of the total 16S rRNA gene in bacteria [47] and the 16S rRNA gene of the genus *Lactobacillus* [48] were determined in each sample using qPCR according to the instructions. Standard curves were generated using serial tenfold dilutions of plasmid standard containing the target fragment. The range of amplification efficiency for the qPCR was from 90 to 110%, and linearity values were all ≥ 0.99. The specificity of the amplification was performed by melting curve analysis and gel electrophoresis. The relative copy numbers of three replicates in each sample was evaluated for each target organism.

### HPV genotyping

HPV testing wasperformed using nucleic acid typing (23 type) testing kit (fluorescence PCR method). Firstly, extracting DNA from the sample, then amplifying the amount of DNA by PCR amplification reaction. The fluorescence signal generated by the fluorescence probe continuously accumulates during the amplification reaction, and the results of each amplification cycle wasmonitored in real-time. After the amplification reaction completed, the results could be judged through the curve of fluorescence signal accumulation. The Ct value of globin in the Cy5 fluorescence detection channel in the sample is ≤ 40, while the Ct value of other fluorescence detection channels shows Undet, indicating a negative result. If the Ct value is ≤ 40, indicating a positive result. The reaction system is divided into 6 reaction tubes, which use four fluorescence detection channels of the instrument for detecting the genotype of 23 human papillomavirus genotypes, as well as the intracellular control β-Global DNA testing. Intracellular control DNA was used to evaluate sample quality and PCR inhibitory factors.

### Measurement of cytokine concentrations

The concentrations of interleukins (IL2, IL4, IL6, IL8, IL10, IL12P70, and IL23), INF-β, and TNF-α were detected using ELISA kit (Shanghai Enzyme-Linked Biotechnology Co., Ltd., China). The operation method was carried out according to the procedure provided by the manufacturer’s instruction with minor modification. The absorbance (OD value) was measured using an enzyme-linked immunosorbent assay (ELISA) at a wavelength of 450 nm. We calculated the content of various cytokines in the sample using a standard curve. Each serum sample should be tested for three times, and the intra batch and inter batch coefficients of variation should be less than 10% and 15%, respectively.

## Result

### Study participant characteristics: HPV positive and HPV negative

In this study, we collected cervical, vaginal, urethral, and rectal samples from 102 subjects, obtaining a total of 408 DNA samples for microbial diversity detection, 17 samples did not produce PCR bands, which did not meet the sequencing requirements. Finally, 391 samples were successfully sequenced. We tested all samples for HPV, and found that the cervical HPV infection rate was not the highest (15.5%), whereas the vaginal HPV infection rate was the highest (33.3%), followed by urethra (29.7%) and cervix, and the rectal HPV infection rate was the lowest (13.1%). Moreover, it was found that most subjects were infected with HPV in multiple sites simultaneously (Additional file 1: Fig. S1A). Molecular typing results showed that the detection rates of HPV-18, HPV-44, HPV-81 and HPV-16 subtypes in the cervix were the highest, the detection rates of HPV16, HPV81 and HPV44 subtypes in the vagina were the highest, and the detection rates of HPV44 subtypes in the urethra were the highest, and the detection rates of HPV44, HPV53 and HPV68 in the rectum were the highest. The detection rate of HPV52 subtype was the highest (Additional file 1: Fig. S1B). Table 1 showed the general characteristics of women with and without HPV infection. Basic variables such as age, BMI, pH and number of pregnancies were not significantly different among the groups. Most women in groups did not smoke (94.1% in the HPV-P group and 98% in the HPV-N group). Non-HPV-infected subjects had more menstrual cycle (68.6% HPV-P vs. 72.5% HPV-N) and more frequent vaginal douching (62.7% HPV-P vs. 70.6% HPV-N).

**Table 1:** Demographics of participants

Characteristics	HPV-P (N = 51)	HPV-N (N = 51)	P values
Mean age (year)	40.9 ± 7.0	42.7 ± 10.3	0.248
BMI (kg/m^2^)	25.5 ± 4.0	24.9 ± 3.4	0.433
PH(Vagina)	4.3 ± 1.2	4.5 ± 1.4	0.488
Educational level (%)			0.946
Non-educated	10 (19.6)	8 (15.7)	–
Primary school	3 (5.9)	3 (5.9)	–
Middle school	11 (21.6)	10 (19.6)	–
Bachelor	19 (37.3)	19 (37.3)	–
≥ Master	8 (15.7)	11 (21.6)	–
Monthly income (¥) (%)			0.367
< 3000	16 (31.4)	13 (25.5)	–
3000–5000	14 (27.5)	10 (19.6)	–
5000–8000	11 (21.6)	19 (37.3)	–
8000–10000	10 (19.6)	9 (17.6)	–
Occupation (%)			0.760
Brainwork	18 (35.3)	18 (35.3)	–
Manual labour	19 (37.3)	16 (31.4)	–
Combination of both	14 (27.5)	17 (33.3)	–
Smoking or not (%)			0.308
Yes	3 (5.9)	1 (2.0)	–
No	48 (94.1)	50 (98)	–
Number of pregnancies	2.7 ± 1.6	2.5 ± 1.3	0.67
Number of abortion (%)			0.443
No	20 (39.2)	14 (27.5)	–
1 times	16 (31.4)	20 (39.2)	–
≥ 2 times	15 (29.4)	17 (33.3)	–
Menstrual period (%)			0.663
Regularity	35 (68.6)	37 (72.5)	–
Irregularity	16 (31.4)	14 (27.5)	–
Vaginal douching (%)			0.401
Yes	32 (62.7)	36 (70.6)	–
No	19 (37.3)	15 (29.4)	–

Data are shown as mean ± SD. *BMI* Body Mass Index

### Distinction in the structure of urogenital tract (cervix, vagina, urethra) and rectal microbial communities due to HPV infection

**Fig. 1:**
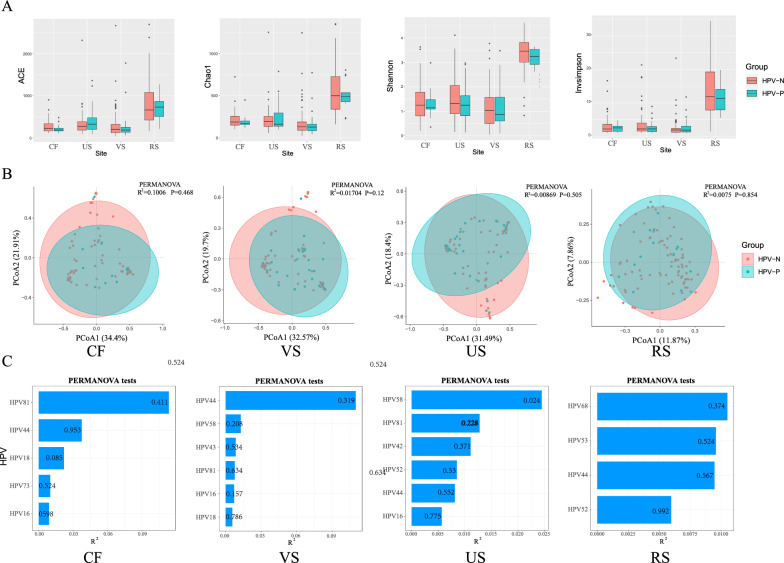
Shifts of microbiota diversity in urogenital tract (cervix, vagina, urethra) and rectal of participants with HPV. **A** ACE, Chao1, Shannon and Invsimpson index of urogenital tract (cervix, vagina, urethra) microbiota and rectal microbiota in participants with or without HPV. **B** PCoA of urogenital tract and rectal microbiota in participants with or without HPV. **C** The effect sizes (R^2^) of HPV types with PERMANOVA tests, the numbers on the bar chart represent P-values

To study the relationship between the microbiota and HPV in different genital areas of women. Firstly, we measured the α diversity of Chao1, ACE, Invsimpson and Shannon indices at different sampling sites of females in the presence or absence of HPV infection. Results showed in Fig. 1, rectal swab had the highest microbial diversity, followed by cervix, urethra and vagina has the lowest diversity. There was no significant difference in α diversity between HPV-positive and HPV-negative groups. PCoA ordination of the microbiota in the four sites showed only a modest separation of the 95% confidence limits of HPV-N and HPV-P groups (Fig. 1B). The PERMANOVA analysis indicated that cervical HPV infection explain 10.06% of the variation in CF microbial community structure (R2 = 10.06% P = 0.468). HPV infection had the lowest effect on rectal microbiota compared to other sites, explaining only 0.75% of the microbiota community structure (P = 0.854) (Fig. 1B). We further analyzed the effects of infection with different HPV subtypes on the microbial community in women at different sampling sites, and found that there were certain differences in the interpretation of the microbial community structure by HPV subtypes in different sites, but most of the differences were not significant (Fig. 1C). In cervix, HPV81 had the greatest effect on microbiota with a PERMANOVA (R2 = 11.35%), followed by HPV44 (R2 = 3.75%) and HPV18 (R2 = 2.20%). HPV44 (R2 = 10.98%) had the greatest effect on the microbiota in the vagina. HPV58 (R2 = 2.44%, P < 0.05) and HPV81(R2 = 1.28%) had the greatest influence on the urethral microbial communities, and HPV58 had a significant effect on the urethral microbial community. It was found that HPV68 (R2 = 10.51%), HPV53 (R2 = 0.96%) and HPV44 (R2 = 0.95%) had the greatest effect on rectal microbiota.

### The composition of urogenital tract and gut microbiota shifts in participants with HPV

**Fig. 2:**
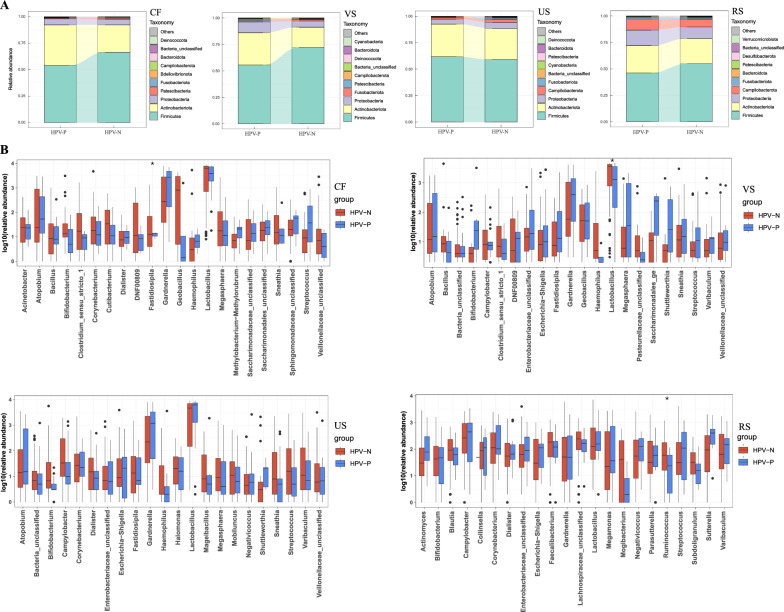
The microbial composition and difference between HPV-P and HPV-N groups. **A** Stacked bar plot of mean proportions of urogenital tract (cervix, vagina, urethra) and rectal derived taxonomic composition between HPV-P and HPV-N groups at phylum level. **B** Statistically differential genera of urogenital tract (cervix, vagina, urethra) and rectal microbiota were evaluated with box plots

The relative abundance at the phylum level showed differences in the urogenital and rectal microbiota without HPV and HPV (Fig. 2A). Firmicute, Actinobacteriota and Proteobacteria were the main dominant bacteria in all the tested parts. In the cervix, vagina and rectum, compared with the HPV-N group, the relative abundance of Firmicutes in the HPV-P group decreased, while the Actinobacteriota and Proteobacteria increased. However, this phenomenon showed an opposite trend in the urethra. Moreover, we further analyzed the composition of the microbiota at the genus level (Fig. 2B). Among the top 22 abundance genera, *Lactobacillus* was still the most dominant in the female urogenital tract, regardless of the HPV infection status, followed by *Gardnerella* and *Atopobium*. The abundance of *Gardnerella* in the reproductive tract was higher in the HPV-P group than in the HPV-N group, while the *Lactobacillus* showed a downward trend. Statistical tests showed that *Fastidiosipila* in cervix, *Lactobacillus* in vagina and *Ruminococcus* in rectum were significantly different in different groups (P < 0.05), and the bacteria genera in these different parts might be affected by or respond to HPV infection. We detected bacterial and *Lactobacillus* specific 16S rRNA genes at different sampling sites by real-time fluorescent quantitative PCR (Additional file 2: Fig. S2). As expected, the number of 16S rRNA genes in total bacteria was higher than in *Lactobacillus*, and the number of 16S rRNA genes in vaginal and rectal swabs was relatively higher. At the same time, HPV infection might affect the bacterial load in different genital tract sites, and increase the number of bacteria and *Lactobacillus* in urethral samples, while no significant differences were observed in cervical, vaginal, and rectal samples (Additional file 2: Fig. S2A, B).

**Fig. 3:**
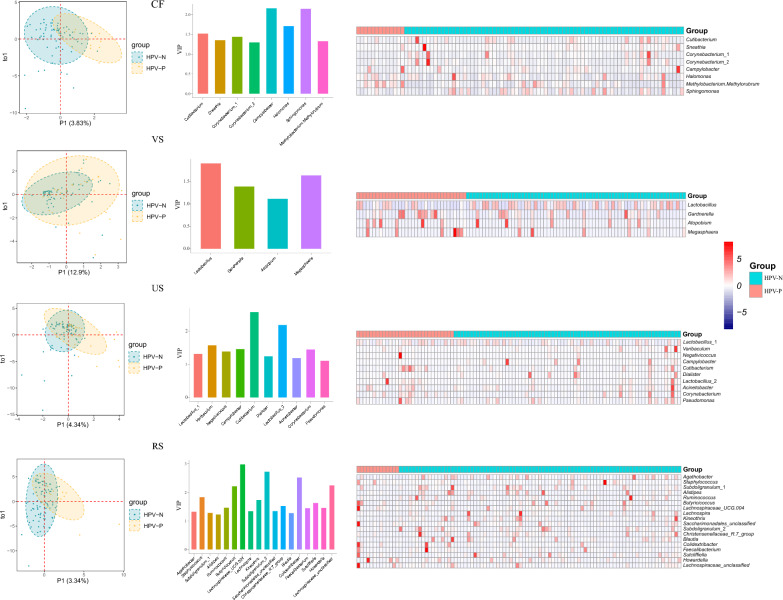
PLS-DA analysis was used to identify the signature microorganisms. **A** Cervical microbiota, **B** vaginal microbiota, **C** urethral microbiota, and (**C**) rectal microbiota. Score scatterplot of PLS-DA model. The Bar chart shows the OTU (VIP value > 1) that significantly promotes the separation of HPV-P and HPV-N groups, and the heat map shows the relative abundance of OTU bacterial genera in each VIP > 1

We then used PLS-DA analysis to further identify microbial markers (VIP > 1) between the HPV-P and HPV-N groups (Fig. 3). The genus levels of 7 (cervix), 4 (vagina), 9 (urethra) and 17 (rectum) OTUs were found in the four sampling sites, respectively, which can differentiate the HPV-P and HPV-N groups. The VIP genera such as *Corynebacterium*, *Lactobacillus*, *Campylobacter*, *Cutibacterium* have been detected at multiple sites, reflecting their possible significance in response to HPV infection across body sites.

### Associations between urogenital tract/rectal microbiota and blood cytokines

We measured serum inflammatory factors, including interleukin-1 (IL-1), interleukin-2 (IL-2), interleukin-4 (IL-4), interleukin-6 (IL-6), interleukin-8 (IL-8), interleukin-10 (IL-10), interleukin-12p70 (IL-12p70), interleukin-23 (IL-23), tumor necrosis factor (TNF)-α, and interferon (IFN)–β using ELISA (Additional file 3: Fig. S3). Overall, leukocyte factor levels increased in the HPV-infected group, among which the levels of IL8 and IL10 in the subjects with cervical HPV infection were significantly increased (P < 0.05), the levels of IL23 in the subjects with urinary tract HPV infection were significantly increased (P < 0.05), and the levels of IFN-β were decreased in the HPV-positive group. The levels of TNF-α in the HPV-P group of CF and RS were higher than those in the HPV-N group.

**Fig. 4:**
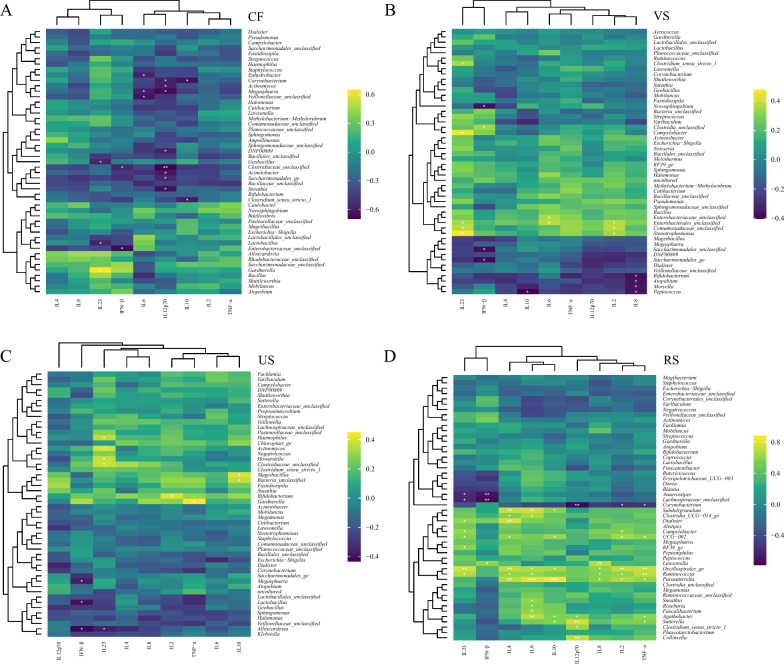
Correlation of microbiota with inflammatory factors. Correlation analysis between the levels of the top 50 genera in the urogenital tract (cervix, vagina, urethra) and rectum and cytokines, *p < 0.05, **p < 0.01, ***p < 0.001

To assess the effect of changes in the lower urogenital tract and rectal microbiota of HPV infection on inflammatory responses, we used Spearman correlation analysis to assess the relationship between serum cytokines and the relative abundance of the top 50 genera in the urogenital tract and rectum (Fig. 4). Levels of IL12p70 in subjects infected with HPV were significantly negatively correlated with most genera, with IL10 associated with *Clostridium_sensu_*stricto_1, *Corynebacterium* showed a significant negative correlation, and it was also found that IL23 had a significant negative correlation with *Lactobacillus* and a significant positive correlation with *Gardnerella* (Fig. 4A). The levels of IL8 in VS HPV-infected subjects were significantly negatively correlated with *Bifidobacterium*, *Atopobium*, *Moryella* and *Peptococcus*. IL23 and IL2 were significantly positively correlated with *Stenotrophomonas*, *Comamonadaceae_*unclassified and *Enterobacterales_*unclassified. At the same time, IL23 was also correlated with *Campylobacter*, There was a significant positive correlation between *Clostridium_sensu_*stricto_1 (Fig. 4B). The *Lactobacillus*, *Alloscardovia*, *Megasphaera* in urethra were significantly negatively correlated with IFN − β. *Gardnerella* was significantly positively correlated with TNF − α. *Haemophilus*, *Actinomyces*, *Howardella*, and *Clostridiaceae_*unclassified was significantly positively correlated with IL23 (Fig. 4C). In terms of rectum, most of the microbiota in the rectum were significantly positively correlated with inflammatory factors. However, we found that *Corynebacterium* was negatively correlated with most inflammatory factors, and its negative correlation with IL12p70, IL2 and TNF − α was statistically significant (Fig. 4D).

We had built association inflammatory factors with VIP genera identified at various sites to further explore the potential association. In the cervical part *Halomonas* and *Sphingomonas* most belongs to the inflammatory cytokines (IL2, IL4, IL6, IL8, IL10, and IL12p70) has significant correlation, IL23 was associated with a significant *Halomonas* only, at the same time, it is interesting to note that there was a significant positive correlation between the two genera (Additional file 4: Fig. S4A). However, the VIP genus in the vagina had no significant association with inflammatory factors (Additional file 4: Fig. S4B). The *Varibaculum* in urethra was significantly associated with IL8 (Additional file 4: Fig. S4C). Meanwhile, it was noted that *Howardella* and *Sutcliffiella* in rectum were significantly correlated with most inflammatory factors, and TNF-α was significantly correlated with *Staphylococcus* (Additional file 4: Fig. S4D).

### Communications between cervix, vagina, urethra and rectal microbiota in HPV infection

**Fig. 5:**
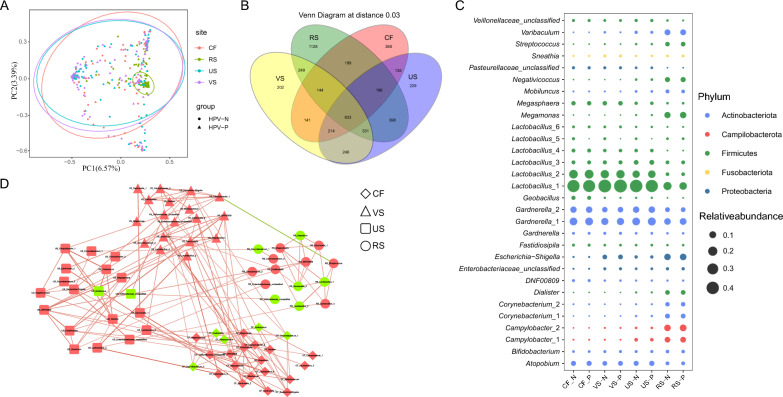
Communications between cervix, vagina, urethra and rectal microbiota in participants with or without HPV. **A** PCoA analysis showed the difference of microbial community structure in different parts of cervix, vagina, urethra and rectal with or without HPV infection. **B** Venn diagrams showed the unique and shared OTUs among cervix, vagina, urethra and rectum. **C** Prevalence and relative abundances of the top 30 shared OTU in no HPV and HPV. **D** Spearman correlation coefficient network diagram of the relative abundance of OTUs shared by the cervix, vagina, urethra, and rectum (Red line: positive correlation and green line: negative correlation, Red nodes: high abundance in the HPV-P group, and green nodes: high abundance in the HPV-N group)

Due to the unique anatomical location of the female reproductive tract, the importance of potential translocation/transmission of microorganisms in disease needs to pay attention. Next, we expanded the analysis to look at potential communication between the subjects' cervix, vagina, urethra, and rectum microbiota. PCoA showed no significant separation between women's cervical, vaginal, and urethral samples, and no differences in microbiome regardless of HPV infection. The 95% confidence ellipse shows that the rectum has a distinct microbiota from the urogenital tract (Fig. 5A). We compared the microbiota of different types of samples and found 833 common OTUs that co-exist in the cervix, vagina, urethra, and rectum by a Venn diagram (Fig. 5B). The relative abundance of the top 30 shared OTUs showed the distribution of major microorganisms between urogenital and rectal samples (Fig. 5C). *Lactobacillus* and *Gardnerella* were the most common bacteria genera in the female cervix, vagina, and urethra, but there was no significant change in the abundance of HPV infection, while the abundance of microorganisms in the rectum was more well-distributed. Then, we conducted correlation analysis for the top 30 shared genera among the 4 different sample sites of females. Overall, cervix, vagina, and urethra had a solid association network, with obvious positive OTU interactions among each other, while rectal microbes communicated less with the other three sites (Fig. 5D). We further analyzed the influence of vaginal microbiome on different adjacent parts (cervix, urethra, rectum) by using SourceTracker. We found these data was consistent with the correlation analysis, cervix was the main potential source of vaginal microbiome, while rectal microbiome had little influence on vagina. Moreover, HPV infection tended to decrease the proportion of different site sources (Additional file 5: Fig. S5). We extracted the first three primary different subclusters by clustering methods, and further viewed a group of OTUs with high correlation (Additional file 6: Fig. S6A). Cluster 1 consists of eight OTUs, six from the cervix (*Megasphaera*, *Mobiluncus*, *Atopobium*, *Fastidiosipila*, *DNF00809*, and *Gardnerella*_2), One *Gardnerella*_2 from the vagina and one *Gardnerella*_2 from the urethra were composed. Among them, *Gardnerella*_2 in the cervix interacts with most OTUs, and was associated with *Gardnerella*_2 in the vagina and urethra. Cluster 2 consists mainly of seven vaginal OTUs and one *Escherichia-Shigella* from the cervix. Cluster 3 contains 4 vaginal OTUs and 3 urethral OTUs. *Fastidiosipila* form vagina was positively correlated with other OTUs. The OTUs of all clusters were grouped together in positive correlation, and the same OTUs of different sites interact with each other. In the correlation network, we further found that the same OTU, including *Lactobacillus*_4 (Additional file 6: Fig. S6B), *Gardnerella*_1 (Additional file 6: Fig. S6C) and *Lactobacillus*_2 (Additional file 6: Fig. S6D) in cervix, vagina and urethra, was correlated with each other in three different parts. It indicated that the translocation/transmission of microorganisms in the female urogenital tract is universal, and their interaction may affect the microbial composition of different ecological niches, which may have an impact on the infection and clearance of HPV in the female genital tract.

### Microbiota discriminate between HPV-P and HPV-N with high accuracy

**Fig. 6:**
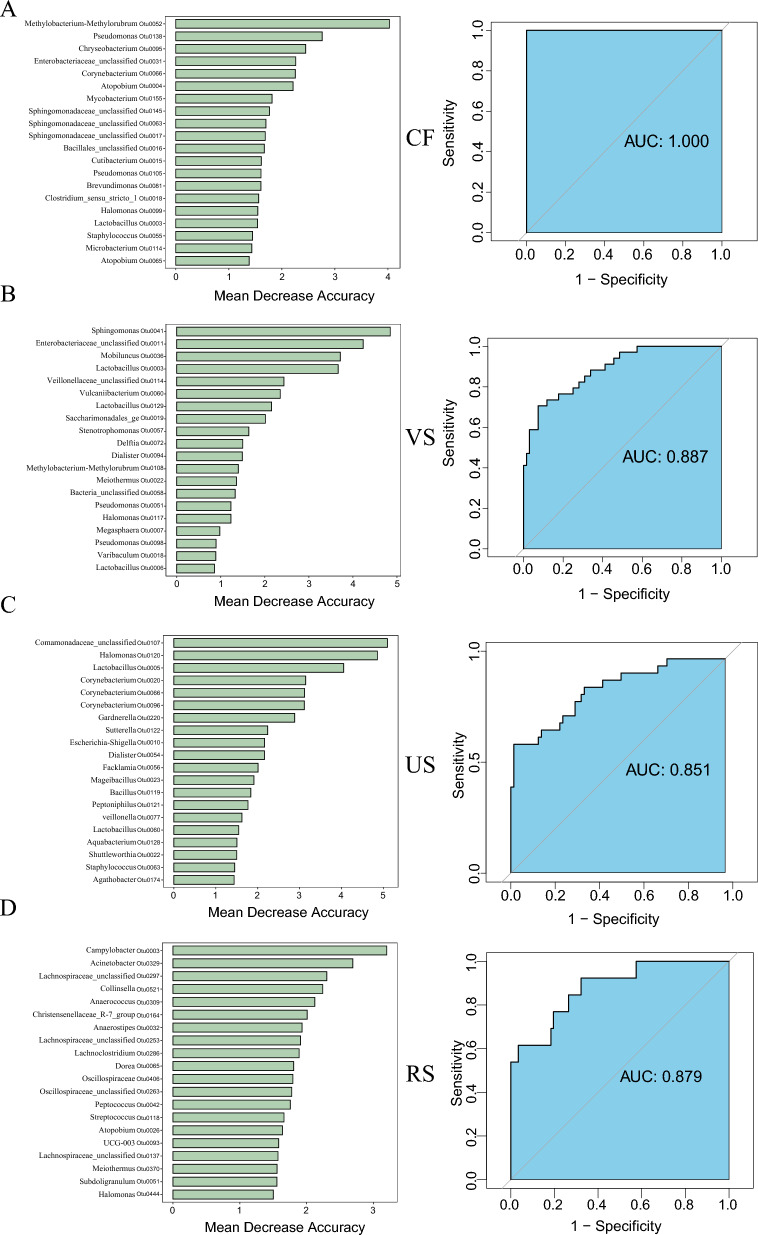
Identify the biomarkers of HPV infected microorganisms based on the presence of HPV in different parts of the body by random forest models. Identify microbial biomarkers, construct a Random forest model to distinguish between two groups of HPV-P and HPV-N, and use the two groups of microbial biomarkers to construct AUC of the optimized model. **A** Cervix (top 20 important species); **B** Vagina (top 20 important species); **C** Urethra (top 20 important species); **D** rectum (top 20 important species)

We explored whether microorganisms could be used to distinguish between HPV-N and HPV-P at different sites by using random forest analysis. We identified several of the most important species that can be considered potential biomarkers to distinguish HPV infection (Fig. 6). We also calculated the predictive value of the combined markers at different sites for the presence or absence of HPV infection, and the receiver operating characteristic (ROC) curve of the optimized microbial marker combination could significantly differentiate the cervix (AUC, 1) (Fig. 6A) from the vagina (AUC, 0.887) (Fig. 6B). HPV-N group and HPV-P group in different parts of urethra (AUC, 0.851) (Fig. 6C) and rectum (AUC, 0.879) (Fig. 6D).

### Pathway analysis

We predicted the microbial metabolic pathways of all samples by picrust2 based on the MetaCyc database. We found the metabolic potential changes in the urogenital tract and intestinal microbiome of women infected with HPV by LEfSe analysis. Three metabolic pathways in the cervix were significantly enriched in HPV-P group (LDA > 2), namely, PENTOSE_P_PWY: pentose phosphate pathway, PWY0_1415: super-pathway of heme biosynthesis from uroporphyrinogen-III, PWY0_1533: methyl-phosphonate degradation I (Additional file 7: Fig. S7A). There were 12 metabolic pathways in the vagina that are significantly different between the HPV-P and HPV-N groups, such as: PWY_6163: chorismate biosynthesis from 3-dehydroquinate, ARO_PWY:chorismate biosynthesis I, COMPLETE_ARO_PWY: super-pathway of aromatic amino acid biosynthesis, PWY0_1061: super-pathway of L-alanine biosynthesis were enriched in HPV-P group. PWY4FS_7: phosphatidylglycerol biosynthesis I (plastidic), PWY4FS_8: phosphatidylglycerol biosynthesis II (non-plastidic), COA_PWY: coenzyme A biosynthesis I, PHOSLIPSYN_PWY: superpathway of phospholipid biosynthesis I (bacteria) were found to be enriched in the HPV-N group (Additional file 7: Fig S7B). Enrichment of PWY_6572: chondroitin sulfate degradation I (bacterial) was found in the urethra of the HPV-P group, while enrichment of multiple isoleucine synthesis pathways in the urethra microflora was detected in the HPV-N group (Additional file 7: Fig. S7C). HEXITOLDEGSUPER_pwy: super-pathway of hexitol degradation (bacteria) and PRPP_PWY: super-pathway of histidine, purine, and pyrimidine biosynthesis were detected to be enriched in the intestinal flora of the HPV-P group, while PWY0_1319: CDP-diacylglycerol biosynthesis II, PWY_5667:CDP-diacylglycerol biosynthesis I, GLYCOGENSYNTH_pwy: glycogen biosynthesis I (from ADP-D-Glucose), PYRIDNUCSYN_pwy: NAD biosynthesis I (from aspartate) were enriched in HPV-N group (Additional file 7: Fig. S7D). These results suggested that HPV infection may indirectly lead to significant differences in the production of precursor metabolites and the biosynthesis of amino acids in the female reproductive tract and rectum.

## Discussion

HPV infection is the most common sexually transmitted infection in women, which is an important factor leading to the occurrence and development of cervical cancer and may also affect female fertility [49, 50]. Women's biological susceptibility HPV and ability to clear HPV might be affected by their own reproductive tract microorganisms. HPV infection might also affect the balance of female reproductive tract microbial communities [51, 52]. it suggested that reproductive tract ecological disorders and/or specific bacteria and cytokines may play a role in HPV infection and the occurrence and/or progression of cervical intraepithelial neoplasia (CIN), and affect the occurrence and development of cervical cancer [53, 54]. However, few studies have conducted a conjoint analysis on the dynamic changes of microorganisms in multiple parts of a female urogenital tract and rectum after HPV infection. In this study, we comprehensively analyze the cervical, vaginal, urinary and rectal microbiota of HPV infected and uninfected female by using 16S rRNA gene sequencing method. Due to the characteristics of women's physiological structure, contamination problems could not be completely avoided. We have regulated sampling operations to the maximum extent possible to minimize the potential for contamination. Although the bacterial interference between original sites due to sampling contamination cannot be completely avoided, we still got exciting and scientifically relevant conclusions. Our data reveals differences in microbial diversity and composition between HPV infection and non-infection in the asymptomatic female reproductive tract and rectum. We built potential connection between microorganisms in different parts and inflammatory factors.

About 5% of all cervical cancer in the worldwide are attributable mainly to those known as high-risk, including HPV types 16, 18, 31, 33, 35, 39, 45, 51, 52, 56, 58, and 59 [55]. In this study, in addition to the common high-risk types such as HPV16, HPV18 and HPV58 detected, we also found that low-risk types such as HPV44 and HPV81 present in multiple sites with high infection rates, it may have a certain impact on the composition of the microbial community in the female urogenital tract. In one of our previous studies, we found that HPV detected in cervical swabs is high-risk, while low-risk HPV is mainly found in vaginal and rectal swabs, and multiple subtypes of infection are common [38]. Some studies have found that HPV44 is one of the common types in tumor tissues of cervical cancer patients [56], frequently detected in human intestines [57, 58]. Studies have shown that HPV81 is detected in the majority of women with cervical cytological abnormalities and is associated with precancerous and cancerous lesions [59, 60]. This study found that HPV subtypes such as HPV44 and HPV81 have significant effects on the microbial communities in different sites, and they may induce the occurrence of female reproductive tract diseases by disrupting the composition of the female reproductive tract microbiota. However, research on the interaction between different HPV subtypes and the reproductive tract microbiota is currently limited, and more experimental exploration is needed to elucidate the mechanisms underlying the interaction between microorganisms and different HPV subtypes.

HPV infection in women have focused on the vagina and cervix, two susceptible sites in ecological studies. Due to the physiological structure of cervix and vagina, vaginal microecological imbalance directly increases the chance of HPV infection and accelerates the process of cervical precancerous lesions [61]. The imbalance of the vaginal microbiota will reduce the stability of the vaginal environment and the resistance to external interference factors, thus affecting the biological susceptibility to HPV acquisition and the immune ability to clear HPV infection [52, 62]. It has been reported that the increase of vaginal microbiota diversity is associated with persistent HPV infection and cervical disease progression [63, 64]. Among the spectrum of microbial species, the female genital tract is mainly dominated by *Lactobacillus* species, which are considered to be one of the simplest yet most important [65]. *Lactobacillus* plays an important role in maintaining environmental health, preventing reproductive tract infections by controlling vaginal pH, reducing glycogen to lactic acid, and stimulating bacterin and hydrogen peroxide [66, 67]. In our study, we found a decrease in the abundance of *Lactobacillus* in female who infected with HPV in reproductive tract, especially the vagina, might respond to HPV infection. *Gardnerella* is a major pathogen involved in the disease process in the female reproductive tract [68], and a major biomarker of persistent HPV infection [17]. Highly diverse bacterial communities dominated by *Gardnerella* associated with host epithelial barrier disruption and enhanced immune activation [69]. Abnormal changes in *Gardnerella* may promote the colonization of HPV in the epithelium of the cervix. We detected more *Gardnerella* sequences in different parts of female infected with HPV, and *Gardnerella* might have a positive interaction with HPV infection. In addition to the common *Lactobacillus* and *Gardnerella* in the reproductive tract, we also found that *Corynebacterium*, *Campylobacter*, *Cutibacterium* and other bacteria genera associated with HPV infection in multiple parts of the female urogenital tract. As a common pathogen in human urogenital tract, *Corynebacterium* is mostly associated with urinary tract infection [70, 71]. Meanwhile, previous studies have found that HPV infection is accompanied by the change of the abundance of *Corynebacterium* [72, 73]. Some studies have listed *Campylobacter* as a marker bacterium of cervical cancer [74], and *Campylobacter* is associated with HPV infection. Some studies have detected a high abundance of *Campylobacter* in the oral cavity infected with HPV [75], suggesting that *Campylobacter* might be involved in the colonization of HPV in the oral cavity. As a kind of skin-borne bacteria, the abundance of *Cutibacterium* in reproductive tract is correlated with HPV infection. Studies have found that the concentration of *Cutibacterium* in reproductive tract of patients treated with HPV has decreased [76]. It also indicated that *Cutibacterium* might have a positive correlation on HPV infection.

Microbial migration and translocation between different parts of the reproductive tract have been proposed, and increasing infection is the main mode of transmission of pathogenic microorganisms in the female reproductive tract [77, 78]. In a study of reproductive tract microbes of women with CIN, it was found that the microorganisms with the most significant changes under the influence of CIN, which had a good agreement between cervix and vagina [79]. Firstly, significant associations between vaginal and urinary microbiomes were also demonstrated, with *Lactobacillus* being predominant in both urine and vagina [80]. Secondly, rectal microbes have extensive microbes communication in female reproductive tract. study have suggested that fecal transplantation may also be an innovative treatment option for female reproductive tract diseases based on specific gut bacterial patterns [21]. In this study, we have found a solid co-occurrence network between the cervix, vagina and urethra, in which the vaginal microbiome was the most interactive with the rest of the site in female and was most influenced by the cervical microbiome. This was consistent with previous studies, cervix and vagina have certain similarities in their microbial composition [30, 38]. At the same time, we found that *Lactobacillus* and *Gardnerella*, the two most common probiotics and pathogenic bacteria in the reproductive tract, have positive correlations in three parts of cervix, vagina and urethra, and these two common bacteria genera in the reproductive tract may have cross-site interactions. Therefore, we believed that translocation communication between different sites might be another mechanism for the stability of female urogenital microbiota and resistance to infection by external factors, such as HPV.

The microbiome plays a crucial role in the regulation of the host inflammatory response. To investigate the interaction between the microbiome and inflammatory response in the female reproductive tract and rectum, we performed an integration analysis between bacterial genera and inflammatory factors which were significantly affected by HPV infection. Previous studies have found that cytokine responses are associated with human HPV infection and clearance [81]. Our study also found that HPV infection might affect cytokine content, including IL8, IL10 and IL23 respond strongly to HPV infection. IL8 levels had related to the progression of HPV infection [82], and IL23 expression is associated with the progression of cervical cancer [83]. Our study found that the expression of IL23 is related to *Lactobacillus* and *Gardnerella*, so it might play a role in HPV infection. Inflammatory factors were also associated with several VIP genera under different sites of a female. We found that two VIP bacteria, *Halomonas* and *Sphingomonas*, present in the cervix, which were associated with multiple inflammatory factors. *Sphingomonas* found to be associated with the progression of HPV infection [84], and interaction with inflammatory factors may be one way that *Sphingomonas* is involved in HPV infection. However, there are few reports on the role of *Halomonas* in the female reproductive tract and HPV infection, and its interaction with inflammatory factors and response to HPV infection need further study.

At present, machine learning such as random forest analysis is increasingly applied in the field of medical diagnosis. We found that several microbial combinations of markers can effectively distinguish between HPV-P individuals and HPV-N individuals at different sites by using random forest. These findings suggest that using different microbial species as a combined biomarker group has the potential to diagnose HPV infection. Because all individuals in this study were recruited from the same region, more clinical studies using a larger multi-center sample should be conducted to verify this diagnostic performance before further clinical progress can be made.

Our research has several limitations. Firstly, the size of the small sample might limit our statistical ability for analysis. Secondly, although sample collection was carried out by professional medical personnel, potential contamination could not be completely avoided due to the proximity of sampling sites. Even if we tried to avoid this issue during the sampling process, caution should be exercised when explaining the possibility of microbial translocation between different sites.

In conclusion, the study revealed the microbial changes in the female urogenital tract and rectum under HPV infection, as well as the microbial communication network between different parts of females. The microbial translocation transmission between different parts might affect HPV infection, and the correlation between microorganisms and cytokines in different parts of females might also affect the colonization of HPV infection. The combined analysis of multiple sites might be able to more comprehensively reveal the coping strategies of human microorganisms under HPV infection. Using human microbiota for preventing or even intervening in human diseases might need the microbiome of non-diseased parts, and by intervening in the microbiome of multiple related parts of females, establish a healthy and stable microbiome to cope with the occurrence and development of diseases.

### Supplementary Information

**Additional file 1: Figure S1.** Overview of HPV infection in the cohort population in this study. The HPV infection status in the cervix, vagina, urethra, and rectum of sampling population A, and the proportion of HPV subtypes in the cervix, vagina, urethra, and rectum.

**Additional file 2: Figure S2.** Copy numbers of the total bacterial and *Lactobacillus* 16S rRNA genes measured by quantitative real-time PCR. Comparison of total bacterial (A) and *Lactobacillus* (B) 16S rRNA gene copy numbers between HPV negative and HPV positive samples from cervical, vaginal, urethral, and rectal swabs.

**Additional file 3: Figure S3.** Comparison of cytokine content in different parts with and without HPV infection.

**Additional file 4: Figure S4.** Correlation analysis between the relative abundance of signature OTU at the genus level and cytokines. Based on HPV-P and HPV-N groups, PLS-DA analysis was used to analyze the correlation between important OTUs (VIP value > 1) and cytokines in different parts of the cervix (A), vagina (B), urethra (C) and rectum (D).

**Additional file 5: Figure S5.** SourceTracker to evaluate microbial communication from cervix to vagina, urethra to vagina, and rectum to vagina.

**Additional file 6: Figure S6.** The microbial interaction network between different parts. A. The three sub networks with the highest scores were selected. The interaction between different *Lactobacillus* strains (B, C) in the cervix, vagina, and urethra The interaction between *Gardnerella* (D) in the cervix, vagina, and urethra.

**Additional file 7: Figure S7.** HPV infection leads to changes in microbial function in different parts. Identification of differential functional pathways of microorganisms in the cervix (A), vagina (B), urethra (C), and rectum (D) after HPV infection by LEfSe analysis.

## Data Availability

The PCR product sequencing data of the article entitled “Changes in microbial composition and interaction patterns of female urogenital tract and rectum in response to HPV infection” had been uploaded to The National Omics Data Encyclopedia at https://www.biosino.org/node/search, accession number: OEP004263.
