# Supplementary figures and images for: Changes in microbial composition and interaction patterns of female urogenital tract and rectum in response to HPV infection
Source: J Transl Med. 2024 Feb 1;22:125. doi: 10.1186/s12967-024-04916-2 (PMC10832222; doi:10.1186/s12967-024-04916-2)

A

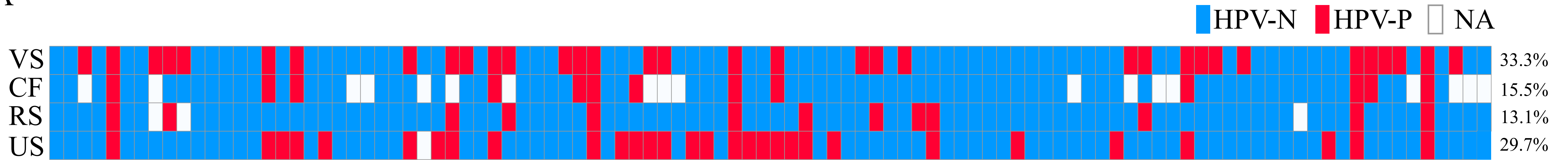

B

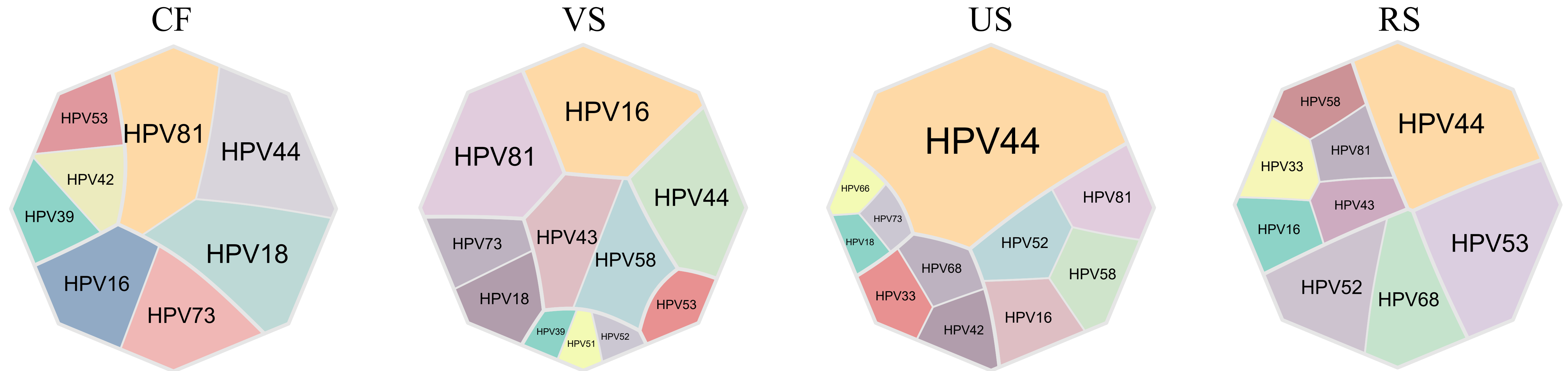

Supplement: Supplementary file 1 — Additional file 1: Figure S1. Overview of HPV infection in the cohort population in this study. The HPV infection status in the cervix, vagina, urethra, and rectum of sampling population A, and the proportion of HPV subtypes in the cervix, vagina, urethra, and rectum. [file 12967_2024_4916_MOESM1_ESM.pdf]

A

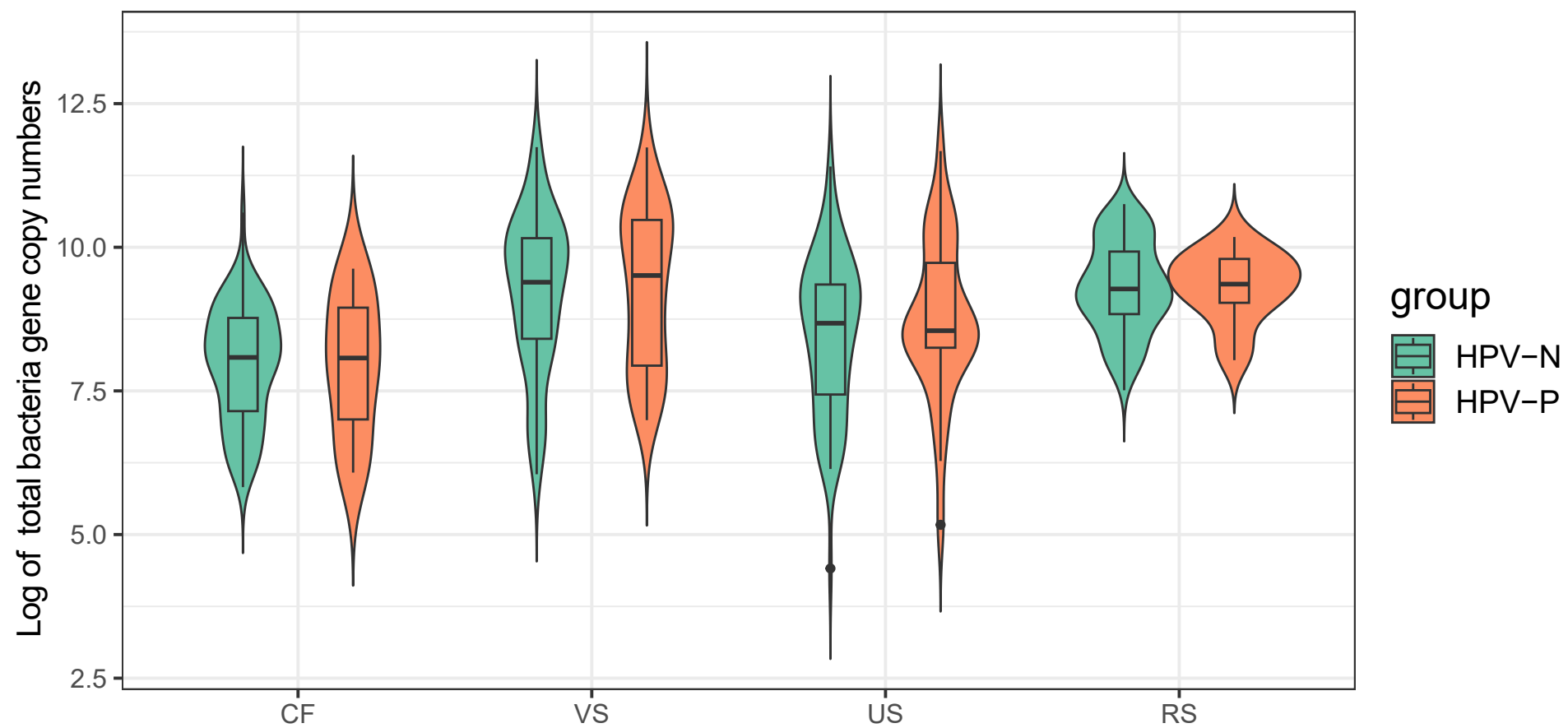

B

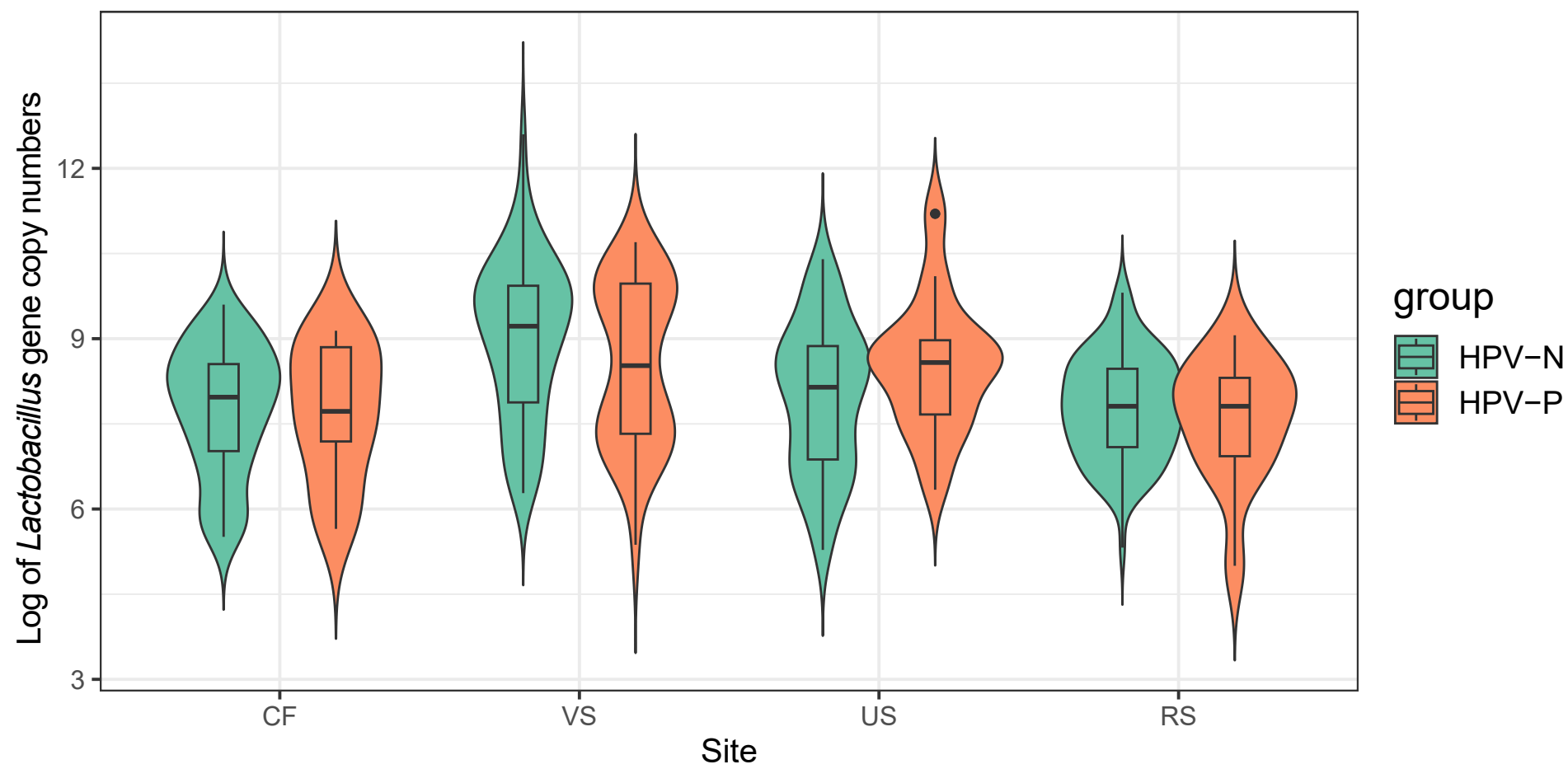

Supplement: Supplementary file 2 — Additional file 2: Figure S2. Copy numbers of the total bacterial and Lactobacillus 16S rRNA genes measured by quantitative real-time PCR. Comparison of total bacterial (A) and Lactobacillus (B) 16S rRNA gene copy numbers between HPV negative and HPV positive samples from cervical, vaginal, urethral, and rectal swabs. [file 12967_2024_4916_MOESM2_ESM.pdf]

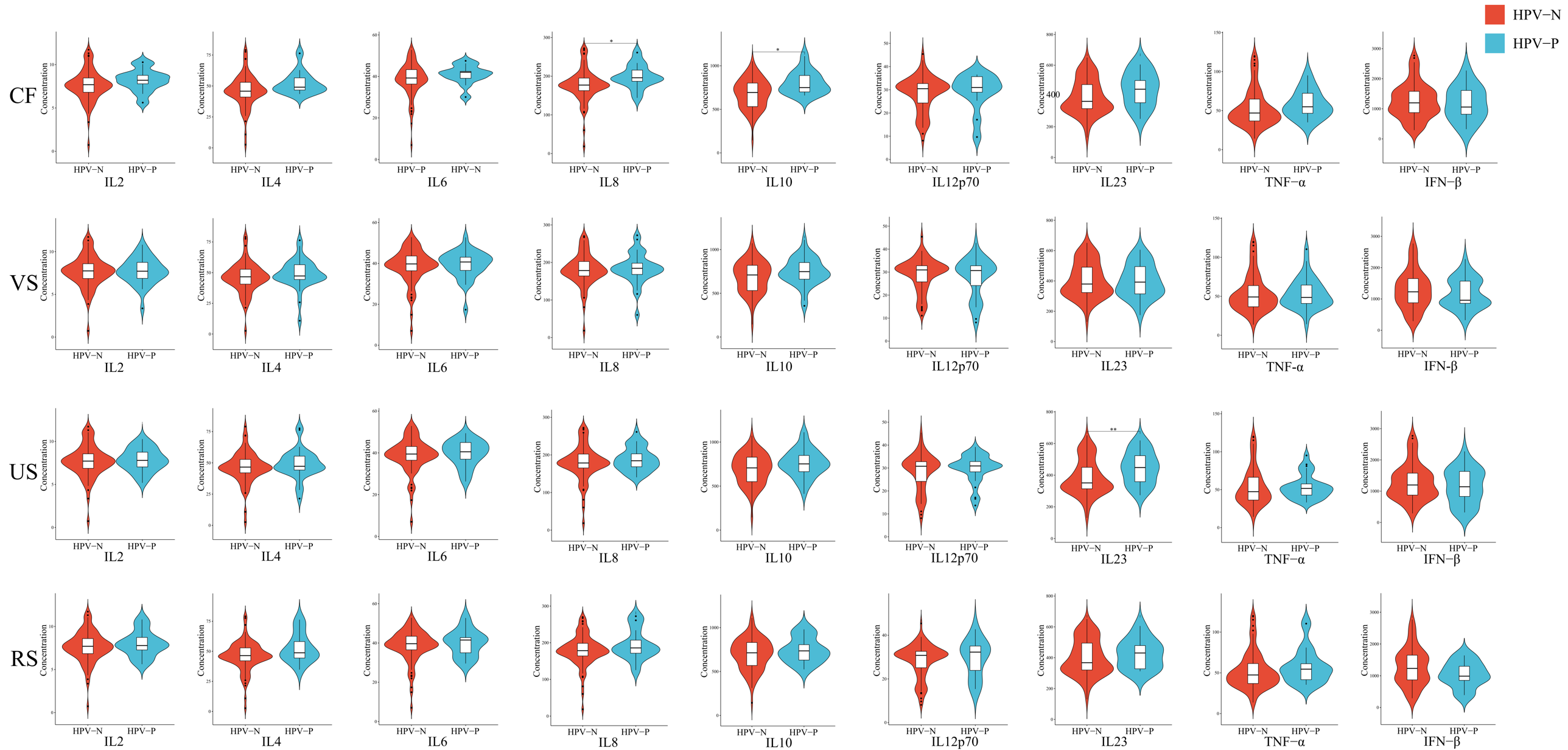

Supplement: Supplementary file 3 — Additional file 3: Figure S3. Comparison of cytokine content in different parts with and without HPV infection. [file 12967_2024_4916_MOESM3_ESM.pdf]

**CF**

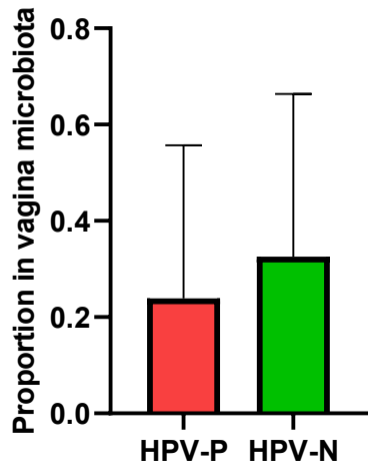

**US**

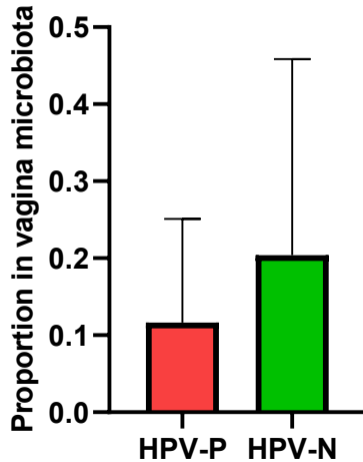

**RS**

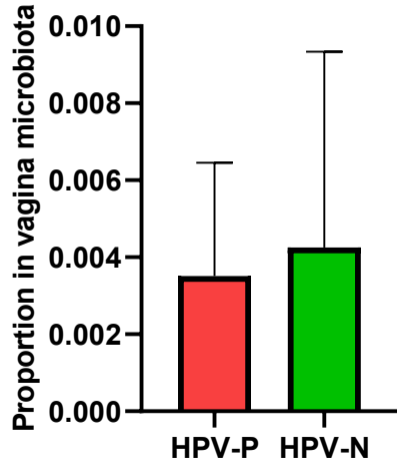

Supplement: Supplementary file 5 — Additional file 5: Figure S5. SourceTracker to evaluate microbial communication from cervix to vagina, urethra to vagina, and rectum to vagina. [file 12967_2024_4916_MOESM5_ESM.pdf]

A

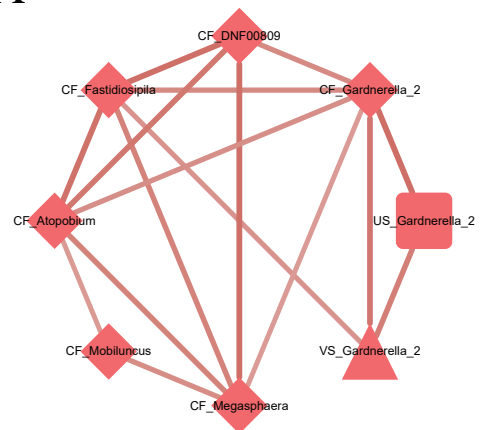

Cluster1

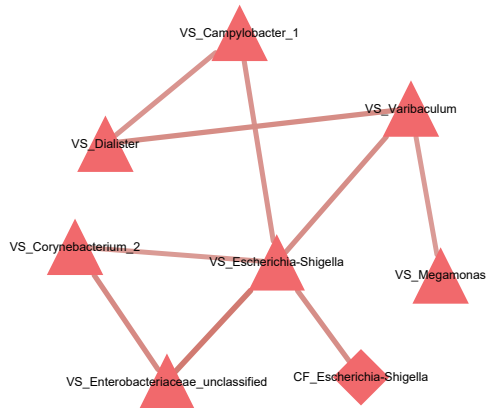

Cluster2

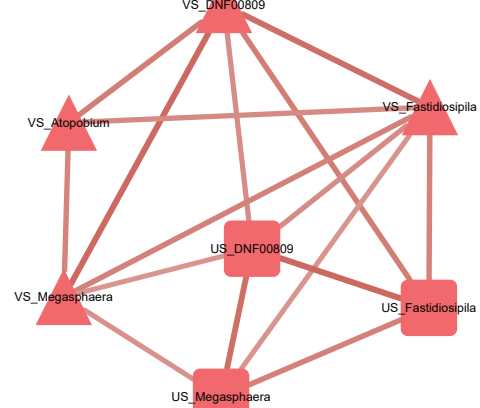

Cluster3

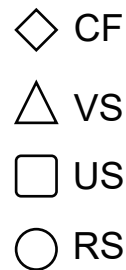

B

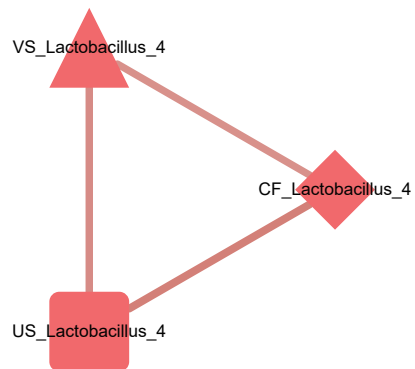

C

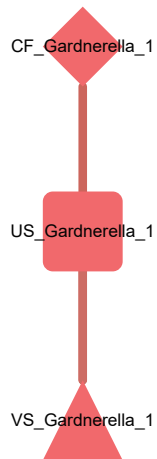

D

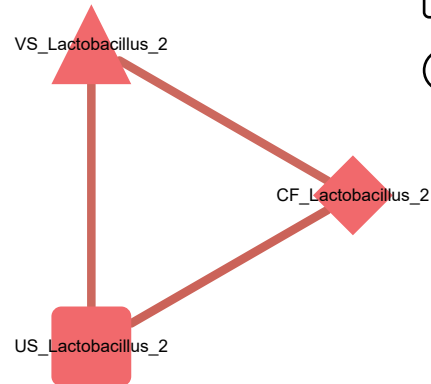

Supplement: Supplementary file 6 — Additional file 6: Figure S6. The microbial interaction network between different parts. A. The three sub networks with the highest scores were selected. The interaction between different Lactobacillus strains (B, C) in the cervix, vagina, and urethra The interaction between Gardnerella (D) in the cervix, vagina, and urethra. [file 12967_2024_4916_MOESM6_ESM.pdf]

A

CF

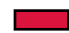 HPV-P
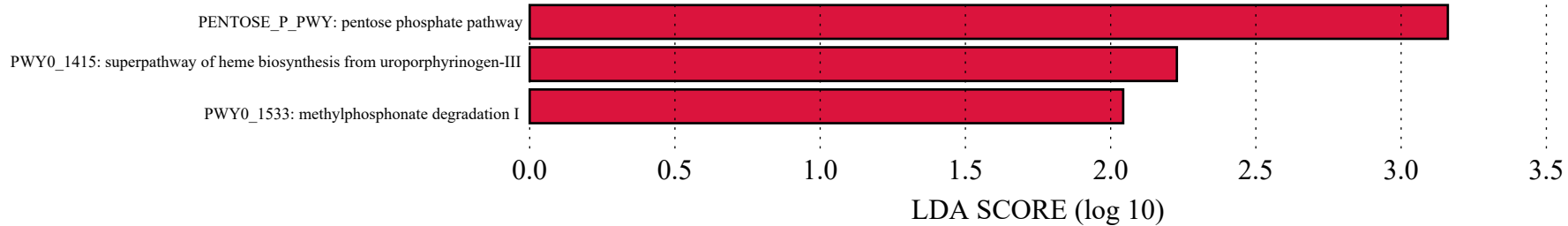

B

VS

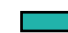 HPV-N

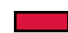 HPV-P
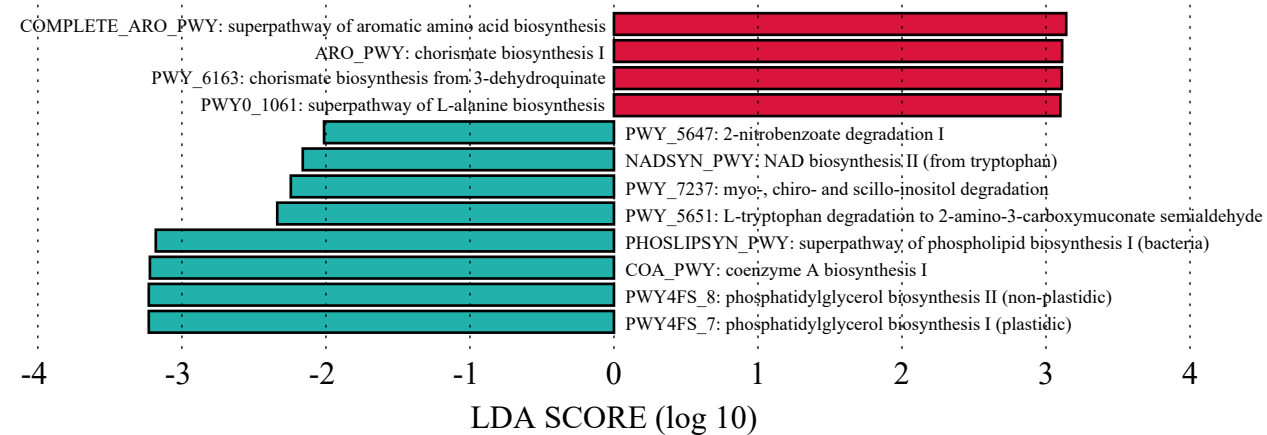

C

US

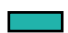 HPV-N

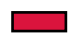 HPV-P
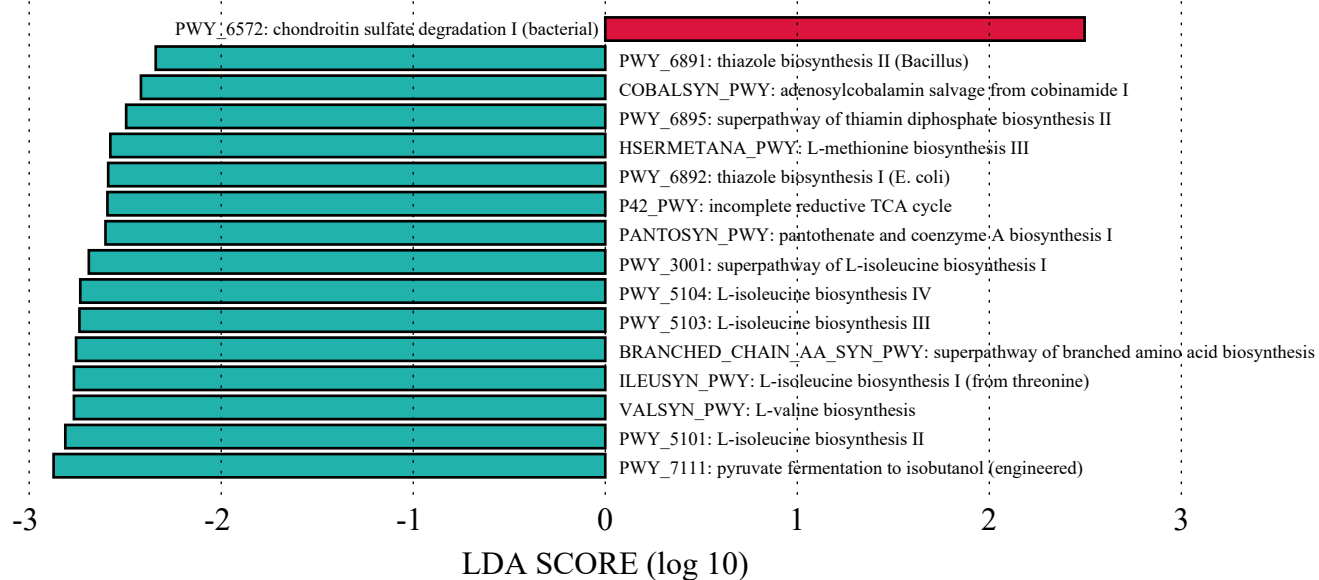

D

RS

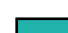 HPV-N

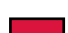 HPV-P
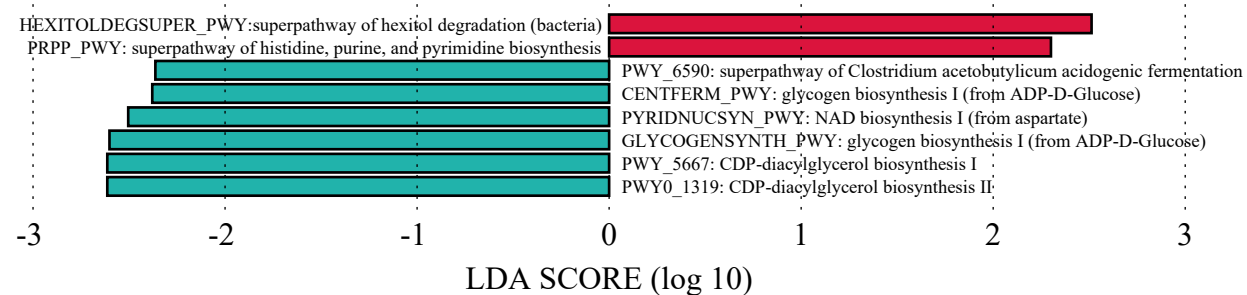

Supplement: Supplementary file 7 — Additional file 7: Figure S7. HPV infection leads to changes in microbial function in different parts. Identification of differential functional pathways of microorganisms in the cervix (A), vagina (B), urethra (C), and rectum (D) after HPV infection by LEfSe analysis. [file 12967_2024_4916_MOESM7_ESM.pdf]
