# Supplementary material for: Changes in microbial composition and interaction patterns of female urogenital tract and rectum in response to HPV infection
Source: J Transl Med. 2024 Feb 1;22:125. doi: 10.1186/s12967-024-04916-2 (PMC10832222; doi:10.1186/s12967-024-04916-2)
Supplement: Supplementary file 4 — Additional file 4: Figure S4. Correlation analysis between the relative abundance of signature OTU at the genus level and cytokines. Based on HPV-P and HPV-N groups, PLS-DA analysis was used to analyze the correlation between important OTUs (VIP value > 1) and cytokines in different parts of the cervix (A), vagina (B), urethra (C) and rectum (D). [file 12967_2024_4916_MOESM4_ESM.pdf]

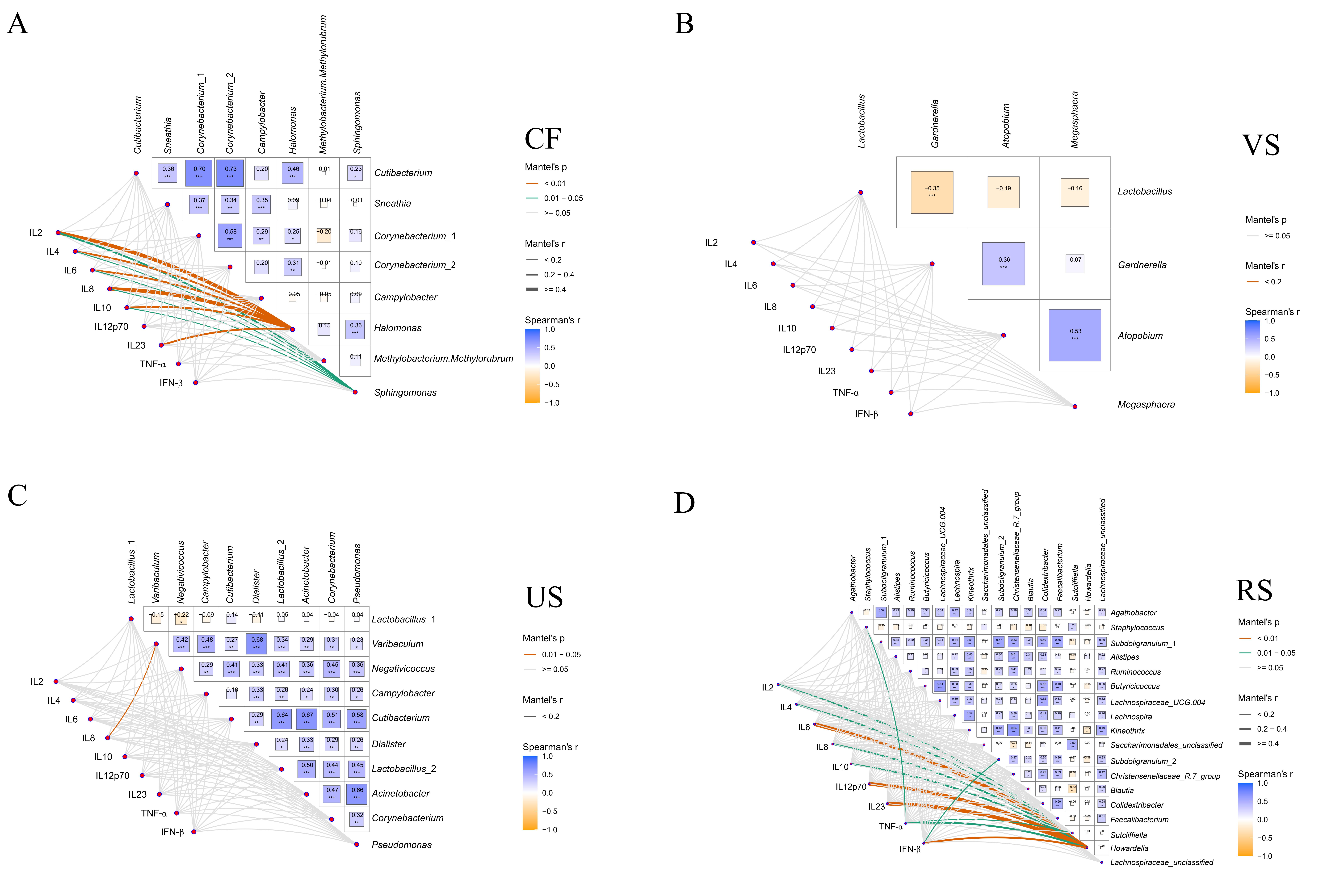
A
Cutibacterium
Sneathia
Corynebacterium_1
Corynebacterium_2
Campylobacter
Halomonas
Methylobacterium.Methylorubrum
Sphingomonas
IL2
IL4
IL6
IL8
IL10
IL12p70
IL23
TNF-α
IFN-β
CF
Mantel's p
< 0.01
0.01 − 0.05
>= 0.05
Mantel's r
< 0.2
0.2 − 0.4
>= 0.4
Spearman's r
1.0
0.5
0.0
−0.5
−1.0
B
Lactobacillus
Gardnerella
Atopobium
Megasphaera
VS
Mantel's p
>= 0.05
Mantel's r
< 0.2
Spearman's r
C
Lactobacillus_1
Varibaculum
Negativicoccus
Campylobacter
Cutibacterium
Dialister
Lactobacillus_2
Acinetobacter
Corynebacterium
Pseudomonas
US
Mantel's p
0.01 − 0.05
>= 0.05
Mantel's r
< 0.2
Spearman's r
D
Agathobacter
Staphylococcus
Subdoligranulum_1
Alistipes
Ruminococcus
Butyricicoccus
Lachnospiraceae_UCG.004
Lachnospira
Kineothrix
Saccharimonadales_unclassified
Subdoligranulum_2
Christensenellaceae_R.7_group
Blautia
Colidextribacter
Faecalibacterium
Sutcliffiella
Howardella
Lachnospiraceae_unclassified
RS
Mantel's p
< 0.01
0.01 − 0.05
>= 0.05
Mantel's r
< 0.2
0.2 − 0.4
>= 0.4
Spearman's r
